# Preparation of Benzothiazolyl-Decorated Nanoliposomes

**DOI:** 10.3390/molecules24081540

**Published:** 2019-04-18

**Authors:** Spyridon Mourtas, Panayiota Christodoulou, Pavlos Klepetsanis, Dimitrios Gatos, Kleomenis Barlos, Sophia G. Antimisiaris

**Affiliations:** 1 Laboratory of Pharmaceutical Technology, Dept. of Pharmacy, School of Health Sciences, University of Patras, 26510 Rio, Greece; pani_christodoulou@hotmail.com (P.C.); klepe@upatras.gr (P.K.); S.Antimisiaris@upatras.gr (S.G.A.); 2 Institute of Chemical Engineering Sciences of the Foundation for Research and Technology Hellas (FORTH/IEC-HT), 26504 Rio Patras, Greece; 3 Department of Chemistry, University of Patras, 26510 Rio Patras, Greece; d.gatos@upatras.gr (D.G.); barlos@cblpatras.gr (K.B.); 4 CBL-Patras, Patras Industrial Area, Block 1, 25018 Patras, Greece

**Keywords:** amyloid β, targeting, benzothiazoles, functionalization, nanoliposomes

## Abstract

Amyloid β (Aβ) species are considered as potential targets for the development of diagnostics/therapeutics towards Alzheimer’s disease (AD). Nanoliposomes which are decorated with molecules having high affinity for Aβ species may be considered as potential carriers for AD theragnostics. Herein, benzothiazolyl (BTH) decorated nanoliposomes were prepared for the first time, after synthesis of a lipidic BTH derivative (lipid-BTH). The synthetic pathway included acylation of bis(2-aminophenyl) disulfide with palmitic acid or palmitoyl chloride and subsequent reduction of the oxidized dithiol derivative. The liberated thiols were able to cyclize to the corresponding benzothiazolyl derivatives only after acidification of the reaction mixture. Each step of the procedure was monitored by HPLC analysis in order to identify all the important parameters for the formation of the BTH-group. Finally, the optimal methodology was identified, and was applied for the synthesis of the lipid-BTH derivative. BTH-decorated nanoliposomes were then prepared and characterized for physicochemical properties (size distribution, surface charge, physical stability, and membrane integrity during incubation in presence of buffer and plasma proteins). Pegylated BTH-nanoliposomes were demonstrated to have high integrity in the presence of proteins (in comparison to non-peglated ones) justifying their further exploitation as potential theragnostic systems for AD.

## 1. Introduction

Alzheimer’s disease (AD) is a progressive neurodegenerative disorder of the central nervous system (CNS). Several pathological hallmarks of AD have been identified through the years, such as decreased cholinergic neurons and acetylcholine (ACh) levels, plaques caused by aggregation of protein fragments of amyloid-β (Aβ), tangles associated with irregular phosphorylation of tau protein, and inflammation and increased oxidative stress from reactive oxygen species (ROS) [1,2]. Although the exact cause of AD still remains unknown, several approaches aimed at inhibiting disease progression have advanced to clinical trials [3,4,5]. It has been shown that unbalanced Aβ production and clearance results in rising Aβ monomer levels in the brain, promoting the formation of dimers and larger oligomers. Then, the oligomers progressively aggregate to form protofibrils, fibrils, and plaques. Aβ species are neurotoxic, and are considered as one of the major histopathological hallmarks of AD thereby various methodologies and approaches to target the production and/or clearance of such amyloid-beta (Aβ) peptide species are currently considered for therapeutic and/or diagnostic purposes [1,6,7,8,9,10,11,12,13].

Surface functionalization of biocompatible/biodegradable and stealth nanoparticles has been extensively used as a method to increase the bioavailability of nanoparticle-associated drugs and/or increase nanoparticle binding affinity to specific receptors due to multivalency [14,15,16]. Among the known nanoparticle types, nanoliposomes have many advantages for drug delivery applications due to their non-toxic/non-immunogenic, fully biodegradable, and structurally versatile nature [17]. Examples of surface decorated nanoliposomes proposed for AD diagnosis/therapy include phosphatidic acid (PA) and cardiolipin (CL) nanoliposomes which were able to target aggregated forms of Aβ1-42 with high binding affinity (K_D_: 22–60 nM) [18]. Additionally, nanoliposomes decorated with curcumin derivatives demonstrated high affinity for Aβ1-42 fibrils (K_D_: 1–5 nM) and sufficient integrity/stability for in vivo applications [19], while anti-Aβ monoclonal antibody (Aβ-MAb) decorated nanoliposomes demonstrated high affinity towards Aβ monomers and fibrils (with K_D_ values between 0.5 and 2 nM) [20]. Also, nanoliposomes which were decorated with tetracycline derivatives [21] and non-planar curcumin derivatives [19,22,23] were efficient in delaying the aggregation of Aβ peptide monomers.

Taking into consideration that thioflavin-T (ThT) is the most widely used (in vitro) indicator of Aβ aggregation displaying fluorescence enhancement and a characteristic red shift when binding to Aβ aggregates, the decoration of nanoliposomes with benzothiazoles (BTH), the moiety that is responsible for the affinity of ThT towards Aβ aggregates [24,25,26], may potentially be an improved method for Aβ targeting. In fact, benzothiazoles bearing only the 2-benzothiazolyl-moiety [27,28,29,30], or more complicated 2-benzothiazolyl-derivatives [31,32,33,34,35,36], have been prepared and tested for their affinity towards amyloids, however none of the previous molecules were ever tested after immobilization on nanoliposomes. Additionally, it should be pointed out that the bulky and ionic nature of ThT is a negative parameter for its permeation across the blood–brain barrier (BBB), explaining why no benefits were obtained from this compound in vivo [37,38,39,40].

In this context, we focused the current study on the decoration of nanoliposomes with non-charged, less hindered BTH-groups, which have also been proven to possess affinity towards Aβ species [27,28,29,30]. In order to develop methods for efficient nanoliposome functionalization with BTH-groups, we synthesized a BTH lipid-derivative.

## 2. Results and Discussion

### 2.1. Synthesis of Compounds

#### 2.1.1. Optimization of BTH Formation

**Scheme 1:**
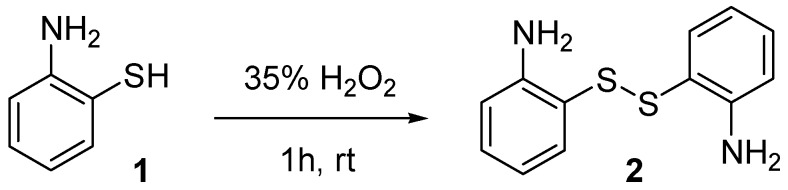
Synthesis of bis(2-aminophenyl) disulfide.

Since the reaction of acyl chlorides with 2-aminobenzenethiol **1** in presence of a base is known to produce by-products [41], we decided to use bis(2-aminophenyl) disulfide **2** for the synthesis of a novel lipid-benzothiazolyl derivative (lipid-BTH). For this, bis(2-aminophenyl) disulfide was initially synthesized, by oxidation of 2-aminothiophenol with 35% hydrogen peroxide (H_2_O_2_) (Scheme 1).

**Scheme 2:**
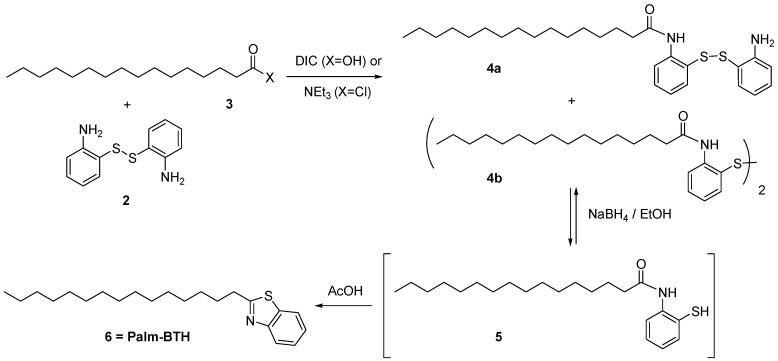
Synthetic method for the formation of Palm-BTH; Compound **3** was used either as palmitic acid (X=OH) when *N*,*N*′-diisopropylcarbodiimide (DIC) was used as a condensing agent, or as palmitoyl chloride (X=Cl) when triethylamine (NEt_3_) was used as a base.

In order to identify the optimal conditions for the preparation of lipid-BTH, we reacted palmitic acid with bis(2-aminophenyl) disulfide and *N*,*N*′-diisopropylcarbodiimide (DIC) as activator of the carboxylic groups; or palmitoyl chloride and triethylamine (NEt_3_) as a HCl scavenger (Scheme 2).

**Figure 1:**
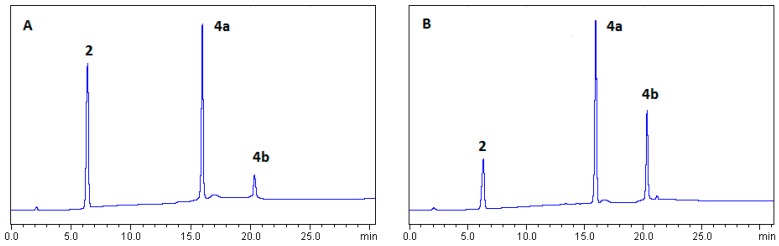
HPLC analysis during the reaction between bis(2-aminophenyl) disulfide and palmitic acid in tetrahydrofuran (THF), using DIC as condensing agent; (**A**) t=1d at rt; (**B**) t=3d at rt; Column: LiChrospher^®^ 100 RP-18 (5 µm) LiChroCART^®^ 250-4; Mobile phase: THF/H_2_O; Gradient: 50% THF to 100% THF in 30 min; Flow rate: 1ml/min; Detection at 254 nm.

**Figure 2:**
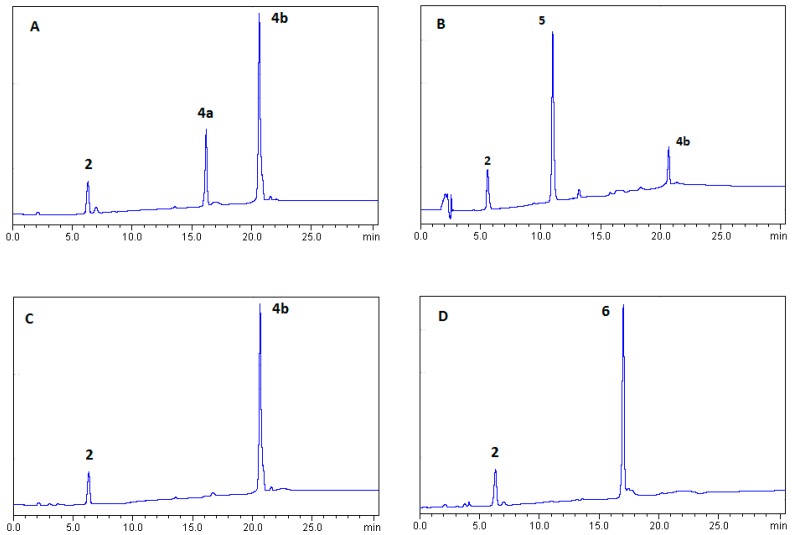
HPLC analysis of (**A**) the final mixture of **4a**+**4b** after 7 days reaction at rt of bis(2-aminophenyl) disulfide and palmitic acid in THF by using DIC as condensing agent; (**B**) during the reduction of **4a**+**4b** with NaBH_4_; (**C**) after the reduction of **4a**+**4b** with NaBH_4_ without subsequent acidification of the mixture; (**D**) after the reduction of **4a**+**4b** with NaBH_4_ and subsequent acidification with AcOH; Column: LiChrospher^®^ 100 RP-18 (5 µm) LiChroCART^®^ 250-4; Mobile phase: THF/H_2_O; Gradient: 50% THF to 100% THF in 30 min; Flow rate: 1mL/min; Detection at 254nm.

The reaction of palmitic acid and bis(2-aminophenyl) disulfide in presence of DIC, formed, as expected, the mono- **4a** and bis-coupled **4b** products. The reduction of the mixture (**4a**+**4b**) was done by the use of NaBH_4_ in EtOH which gave the desired Palm-BTH derivative **6**, after the addition of acetic acid (AcOH) (Scheme 2). In order to identify the best reaction conditions and the conditions that are required for the cyclization of the reduced compound **5**, to the corresponding Palm-BTH **6**, we followed the synthetic procedure by HPLC analysis (Figure 1 and Figure 2). It was found that, the coupling reaction between palmitic acid and bis(2-aminophenyl) disulfide gave a mixture of **4a** (Figure 1; 16.0 min) and **4b** (Figure 1; 20.8 min) products, as expected.

Although the coupling reaction was rather slow, as indicated by HPLC analysis (Figure 1), no by-products were formed, even after 7 days of reaction between **2** and the activated palmitic acid at rt (Figure 2A). The reaction mixture was then reduced with NaBH_4_ in EtOH. HPLC analysis of the reduction mixture proved the existence of the reduced free thiol **5** (Figure 2B; 11 min). It was also proven from the HPLC results that, in the absence of AcOH, the reduced compound **5** does not form the desired BTH-product but isomerizes back to the oxidized **4b** during the isolation process (Figure 2C).

In order to enable cyclization of **5**, we used acetic acid (AcOH), which favors the cyclization of the reduced free thiol group of **5** to the desired Palm-BTH (**6**: Figure 2D; 17 min), after 1h stirring of the acidified mixture at rt. This experiment revealed the importance of using an acid as catalyst, in order to effectively enable the cyclization of 2-*N*-palmitoyl-aminobenzenethiol **5** and the formation of the desired Palm-BTH **6** [42,43]. Herein we selected AcOH, which is an inexpensive, readily available and easily handled reagent.

**Figure 3:**
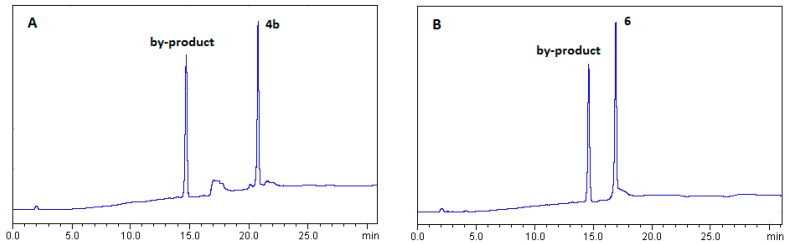
HPLC analysis of (**A**) the reaction mixture product between bis(2-aminophenyl) disulfide and palmitoyl chloride in presence of NEt_3_; (**B**) the reaction mixture after reduction with NaBH_4_ and acidification with AcOH; Column: LiChrospher^®^ 100 RP-18 (5 µm) LiChroCART^®^ 250-4; Mobile phase: THF/H_2_O; Gradient: 50% THF to 100% THF in 30 min; Flow rate: 1mL/min; Detection at 254 nm.

In another effort, the reaction of 2-aminobenzenethiol with palmitoyl chloride in presence of NEt_3_, formed, in addition to the desired bis-coupled product **4b** (Figure 3A: 20.8 min), an unexpected by-product (Figure 3A; 14.5 min). Further reduction of the product mixture with NaBH_4_ and addition of AcOH gave the expected Paml-BTH, after the reduction and cyclization of **4b**, but the by-product was not affected, being present in the final reaction mixture (Figure 3B). Although the by-product was not further analyzed, this is an obvious drawback of this specific method for synthesis of Palm-BTH.

#### 2.1.2. Synthesis of Lipid-BTH

**Scheme 3:**
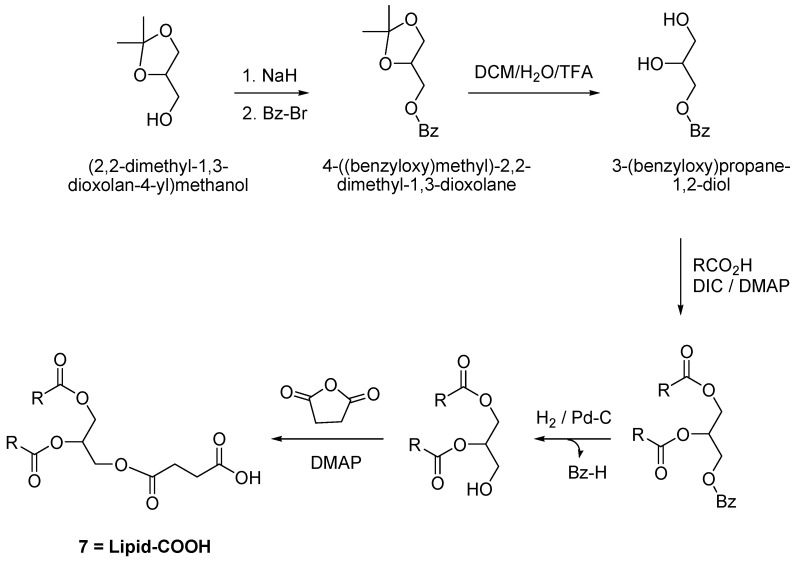
Synthetic method for the formation of Lipid-COOH; R=CH_3_-(CH_2_)_14_-.

The previous observations led us to use a lipid-COOH derivative and to couple it with bis(2-aminophenyl) disulfide, in order to prepare a lipid-BTH derivative. For this, we synthesized the lipid-COOH derivative **7**, according to Scheme 3. In brief, (2,2-dimethyl-1,3-dioxolan-4-yl)methanol was reacted with NaH in methanol (MeOH) and then benzyl bromide (Bz-Br) was added. The derived product was further hydrolyzed in presence of DCM/H_2_O/TFA (10:1:2) to 3-(benzyloxy)propane-1,2-diol, which was further coupled with palmitic acid in presence of DIC and 4-dimethylaminopyridine (DMAP). The *O*,*O*′-bis-acylated product was hydrogenalyzed in presence of Palladium on Carbon catalyst (Pd-C) and finally reacted with succinic anhydride/DMAP to form the desired lipid-COOH derivative **7** (Scheme 3).

**Scheme 4:**
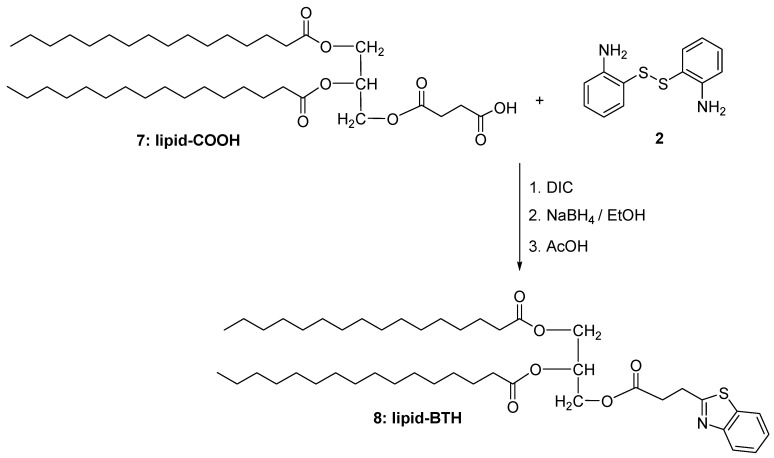
Synthetic method for the formation of lipid-BTH.

**Figure 4:**
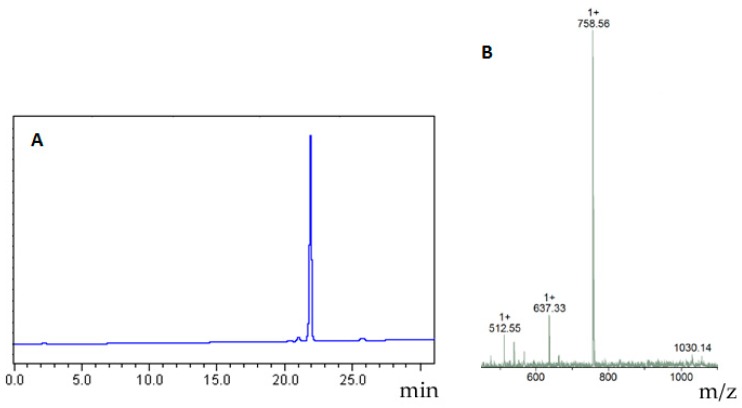
(**A**) HPLC analysis of purified lipid-BTH.; Column: LiChrospher^®^ 100 RP-18 (5 µm) LiChroCART^®^ 250-4; Mobile phase: THF/H_2_O; Gradient: 50% THF to 100% THF in 30 min; Flow rate: 1ml/min; Detection at 254nm; (**B**) ESI-MS of lipid-BTH.

Subsequently, we applied our previous findings for the synthesis of lipid-BTH **8**. Thus, we first performed the coupling reaction of lipid-COOH **7** with bis(2-aminophenyl) disulfide **2** in presence of DIC, and the derived mixture was further reduced with NaBH_4_ in EtOH. This was acidified with AcOH (according to our previous findings) to form the desired lipid-BTH **8** (Scheme 4). The later reaction was monitored by HPLC and our previous findings were confirmed. The derived lipid-BTH was isolated as a white solid and was recrystallized from anhydrous EtOH (and/or AcCN) (total yield: 55%). The product was characterized by HPLC (Figure 4A; 22 min), ESI-MS (Figure 4B; (M+H)^+^: 758.56 (calc.), 758.54 (found)), ^1^H-NMR, ^13^C-NMR (as described in detail in materials and methods).

### 2.2. Nanoliposome Preparation

Following the synthesis of lipid-BTH, we were interested in the preparation of stable (in terms of size distribution, stability, and integrity) nanoliposomes, which could be potentially used for in vivo applications. Thus, the lipid-BTH was incorporated in nanoliposome preparations consisted of six different combinations of 1,2-dipalmitoyl-sn-glycerol-3-phosphatidylcholine (DPPC), Cholesterol (Chol), as well as 1,2-dipalmitoylsn-glycerol-3-phosphatidyl glycerol (DPPG) and 1,2-distearoyl-sn-glycero-3-phosphoethanolamine-*N*-[methoxy(polyethylene glycol)-2000] (ammonium salt) (DSPE-PEG_2000_-OMe (in some cases), by the thin film hydration method. DPPC and Chol were selected in order to prepare nanoliposomes with rigid membranes, and DPPG for formation of negatively charged nanoliposomes [44]; DSPE-PEG_2000_-OMe was used in order to have a PEG coating on the nanoliposome surface, which is known to prolong nanoliposome blood circulation, improve their distribution in tissues, and increase their physical stability (by providing a strong interbilayer repulsion that can overcome the attractive van der Waals forces). A PEG concentration of 8 mole% (to total lipid) was selected in accordance with previous results [44,45].

The exact compositions of the nanoliposomes, as well as their physicochemical properties are presented in Table 1. LIP1 and LIP2 were composed of DPPC/Chol (1:1) and 10% or 20% molar lipid-BTH (for LIP1 and LIP2, respectively). LIP3 and LIP4 were composed of DPPC/DPPG/Chol (9:1:10) with 10% lipid-BTH (LIP3) or 20% lipid-BTH (LIP4). In other nanoliposome types, PEG was inserted in the nanoliposome membranes, and DPPC/Chol/DSPE-PEG_2000_-OMe (1:1:0.08 mole/mole) nanoliposomes were prepared with 10% lipid-BTH (LIP5) or 20% lipid-BTH (LIP6). As seen in Table 1, there is a slight increment in vesicle size as a function of the percent of lipid-BTH incorporation in the membranes, of all the types of nanoliposomes prepared (LIP1 versus LIP2; LIP3 versus LIP4; LIP5 versus LIP6). In all cases the polydispersity index values were relatively low (ranging from 0.148 to 0.186), except for the DPPC/Chol/Lipid-BTH nanoliposomes with 20mol% BTH, which had a PDI value around 0.318, suggesting that these nanoliposomes have an increased tendency to aggregate (compared to all the other nanoliposome types). The ζ-potential of DPPC/Chol and DPPC/Chol/DSPE-PEG_2000_-OMe BTH-decorated vesicles ranges between −2.27 and −4.85, as expected, since all the lipids in their composition are zwitero-ionic; DPPG-containing nanoliposomes have negative ζ-potential values between −9.61 and −12.2 mV, since DPPG is a negatively charged lipid.

**Figure 5:**
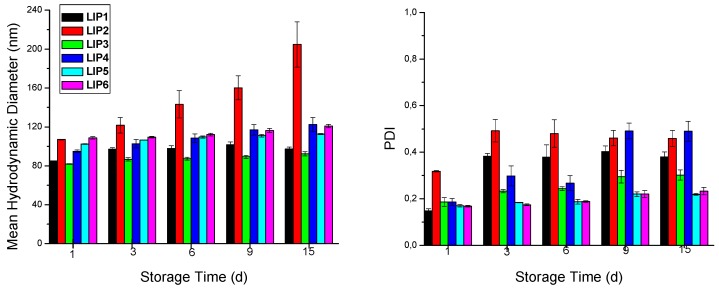
Size (d-hydrodynamic mean diameter, nm) and pdi values of the LIP1–LIP6 prepared dispersions, in PBS buffer 7.4 at a lipid concentration of 5 mg/mL, during storage at 4 °C. Each value is the mean of at least 5 different measurements and bars are the SD values of each mean.

The physical stability (stability of mean hydr. diameter and ζ-potential) of the different nanoliposome preparations during storage at 4 °C, for a period of 15 days, (liposome dispersions with lipid concentration equal to 5 mg/mL were used), are presented in Figure 5. As seen, LIP1 and especially LIP2 nanoliposomes tend to aggregate very fast after their preparation; thereby these specific nanoliposome compositions could not be used for further in vitro/in vivo experiments. The aggregation of LIP1 and LIP2 may be attributed to the BTH groups present on their surface, which may interact either between one another, or with groups of the other lipids, leading to the formation of aggregates. The negatively charged nanoliposomes (with DPPG in their lipid membrane) LIP3 and LIP4 also demonstrate moderate stability during the 15 days storage period. Indeed, the PDI values of LIP 3 increased from 0.186 to 0.295, while for LIP4 the final PDI value was even higher (0.49), possibly due to the higher amount of surface BTH groups on the surface of LIP4 (compared to LIP3). In comparison to LIP1 and LIP2compositions, the DPPG containing nanoliposomes (LIP3 and LIP4) are more stable, demonstrating reduced aggregation during storage, most probably as a result of their surface charge; nevertheless, their PDI values were significantly increased after 9 days (especially in the case of LIP4). Finally, pegylation seems to stabilize BTH-nanoliposomes, since the pegylated LIP5 and LIP6, were found to be very stable (compared to the non-pegylated types), with sizes ranging between 113 and 121 nm (and PDI values around 0.200), after 15 days of storage at 4 °C. Due to their physical stability, the former nanoliposome formulations are the best candidates for further in vitro/in vivo investigations. For all nanoliposome types tested the ζ-potential values did not change during the 15-day storage period.

**Figure 6:**
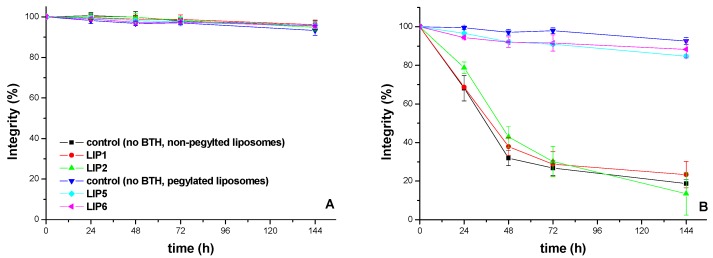
Integrity of non-pegylated BTH-nanoliposomes (LIP1 and LIP2) and pegylated BTH-nanoliposomes (LIP5 and LIP6) and their corresponding control nanoliposomes (with no BTH on their surface), during incubation in buffer (**A**) or FCS (80% *v*/*v*) (**B**) at 37 °C. Each data point is the mean of at least 3 different experiments and the bar is the SD of the mean.

The integrity of the non-pegylated BTH-nanoliposomes LIP1 and LIP2, and of the pegylated BTH-nanoliposomes LIP5 and LIP6 during incubation in presence of buffer (PBS) and serum proteins (FCS) is presented in Figure 6A,B, respectively. Control nanoliposomes (without BTH) are also studied in parallel. As seen, all nanoliposome types were stable during incubation in buffer. The fact that the 10% BTH non-pegylated nanoliposomes LIP1, and the 20% BTH non-pegylated nanoliposomes LIP2, and also the corresponding control nanoliposomes are all equally stable in PBS, indicates that no phase separation or fusion is taking place when lipid-BTH is inserted into the liposomal membrane, at 37 °C, since if this was the case calcein would leak out of the vesicles.

In presence of plasma proteins (FCS), as anticipated due to the absence of PEG, the non-pegylated nanoliposomes (LIP1, LIP2, and corresponding control vesicles) were unstable and their calcein content was observed to rapidly leak out from the vesicles during incubation. On the contrary, both BTH-pegylated nanoliposome types, LIP5 and LIP6, were highly stable in the presence of FCS during the full incubation period of 144 h. Due to their increased integrity under conditions mimicking the in vivo situation (compared to the other BTH-nanoliposome types), the pegylated-BTH-nanoliposomes are the most appropriate to continue studies with, towards the development of potential theragnostic systems for AD [44].

**Figure 7:**
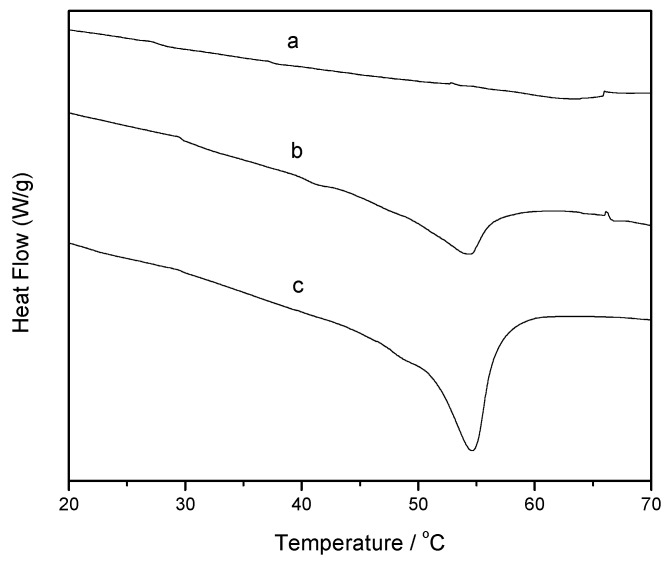
DSC thermographs of control nanoliposomes (no BTH) (a); LIP1 (10% BTH) (b); and LIP2 (20% BTH) (c).

LIP1 and LIP2 as well as control nanoliposomes (without BTH-lipid) were evaluated by Differential Scanning Calorimetry (DSC) in order to understand the effect of BTH-lipid addition in the nanoliposome membrane on their phase transition. As seen in Figure 7, in the case of the control nanoliposomes no transition is observed, which is normal since the transition of DPPC should be abolished due to the presence of Chol [46]. On the contrary, a sharp peak at 54.5 °C is observed in the case of the nanoliposomes that contain lipid-BTH, and the peak recorded follows a lipid-BTH concentration dependent manner. This result provides evidence that the insertion of lipid-BTH in the nanoliposome membrane has an impact on the phase transition of the membrane. In any case no phase-separation relevant results were observed in our other studies, since they were carried out at lower temperatures.

## 3. Materials and Methods

### 3.1. Materials

#### 3.1.1. Synthesis of BTH-Derivatives (Palm-BTH and Lipid-BTH)

All chemicals were purchased from Sigma-Aldrich OM, Athens, Greece, except 2-aminobenzenethiol which was a gift of CBL Patras S.A. (Industrial area of Patras, Building block 1, GR-25018, Patras, Greece). All chemicals were used without further purification, while all required anhydrous solvents were dried with molecular sieves, for at least 24 h prior to use. Thin layer chromatography (TLC) was performed on silica gel 60 F254 plates (Merck, Darmstadt, Germany) and spot detection was carried out by UV light, or by charring with an aqueous solution of K_2_CO_3_/KMnO_4_. Flash column chromatography was performed on silica gel 230–400 mesh (Merck, Darmstadt, Germany). HPLC analysis was performed on a Shimadzu LC-2010 liquid chromatography system (Canby, OR, USA) using a LiChrospher^®^ 100 RP-18 (5 µm) LiChroCART^®^ 250-4; Mobile phase: THF/H_2_O; Gradient: 50% THF to 100% THF in 30 min; Flow rate: 1mL/min; Detection at 254 nm. NMR spectra were recorded at 25 °C with a Brucker DPX 400 MHz instrument (Peoria, IL, USA). Chemical shift assignments, reported in ppm, are referenced to the corresponding solvent peaks. MS were recorded on a QTRAP system with ESI source (Applied Biosystems, Waltham, MA, USA).

#### 3.1.2. Nanoliposome Preparation

1,2-dipalmitoyl-sn-glycerol-3-phosphatidylcholine (DPPC), 1,2-dipalmitoylsn-glycerol-3-phosphatidyl glycerol (DPPG), 1,2-distearoyl-sn-glycero-3-phosphoethanolamine-*N*-[methoxy(polyethylene glycol)-2000] (ammonium salt) (DSPE-PEG_2000_-OMe) were purchased from Avanti Polar Lipids (Alabaster, AL, USA). Cholesterol (Chol), SephadexG50 (course), calcein, Sepharose CL-4B were purchased from Sigma-Aldrich OM, Athens, Greece. All other chemicals were reagent grade. Ultrapure water was produced with a Millipore Direct-Q^®^3 with pump system (Millipore S.A., Molsheim, France). Fluorescence intensity (FI) of samples was measured with a Shimatzu RF-5301PC spectrofluorophotometer (Kyoto, Japan) at 37 ± 0.1 °C. In all cases, 5-nm slits were used. A bath sonicator (Branson 2510E-DTH, Danbury, CT, USA)) and a probe sonicator equipped with a microtip (Vibra-cell, Sonics and Materials, Inc., Newtown, CT, USA) were used for nanoliposome preparation.

### 3.2. Synthetic Procedures

#### 3.2.1. Synthesis of Bis(2-Aminophenyl) Disulfide

20 mL of 2-aminobenzenethiol (187 mmol) were placed into a flask and hydrogen peroxide (H_2_O_2_) 35% was added drop-wise (7mL) at rt in 1h. Then, 150 mL diethylether (Et_2_O) was added and the mixture was extracted, subsequently, with water, aq. NaOH and water. The organic layer was dried with MgSO_4_ and the solvent was evaporated. The resulting yellow crystalline solid was delivered after washing with Hexane (21.4 gr, yield: 92%). ^1^H-NMR (CDCl_3_): 7.21–7.08 (4H, m), 6.80–6.68 (2H, d), 6.65–6.55 (2H, t), and 4.5–4.0 (2H, ds, NH_2_)

#### 3.2.2. Synthesis of Lipid-COOH

Synthesis of 3-Benzyl-sn-Glycerol

We placed 1.271 gr NaH (53 mmol) in a flask. Next, 90 mL THF and 5 gr (37.8 mmol) of 1,2-*O*-isopropylidene-sn-glycerol were added, and the mixture was stirred for 30 min at rt. Then 5.4 mL (45.4 mmol) benzyl bromide were added in 3 portions (in 30 min) and the reaction was stirred overnight at rt. Finally, 40 mL 10% aqueous Na_2_CO_3_ were added and the mixture was stirred for 30 min more at rt. The mixture was then extracted with Et_2_O and the organic phase was washed with H_2_O (×3) and condensed until formation of an oily residue. This was directly hydrolyzed by 20 mL DCM/H_2_O/TFA (10:1:2) for 30 min at rt and the mixture was extracted in Hex and 10% Na_2_CO_3_. The aqueous phase was delivered and washed by Hex (×2). EtOAc was added and the aqueous phase was saturated with NaCl (or brine). The organic phase was delivered, dried with MgSO_4_ and condensed. The oily product was delivered and dried over MgSO_4_ (6.49 gr). Yield: 94%. ^1^H-NMR (CDCl_3_): 7.37–7.28 (5H, m, Ar), 4.54 (2H, s, H4), 3.91–3.87 (1H, m, H2), 3.71–3.59 (2H, 2dd, H1, 1′), 3.58–3.50 (2H, 2dd, H3,3′).


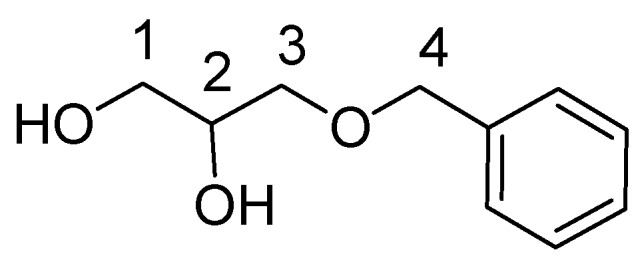


3-Benzyl-1,2-Dipalmitoyl-sn-Glycerol

We placed 6.230 gr (24.294 mmol) of palmitic acid and 3.26 mg (26.72 mmol) DMAP into a flask and these were dissolved in 15 mL DCM and 4ml DMF. Then 4.18 mL DIC (26.72 mmol) were added and the mixture was stirred for 5 min at rt. Following, 2.108 gr (11.568 mmol) 3-benzyl-sn-glycerol were added, and the reaction mixture was stirred overnight. The formed 1,3-diisopropylurea was filtered and the filtrate was extracted in 10% citric acid. The organic phase was washed with H_2_O (×3). Finally the organic phase was dried with MgSO_4_ and condensed. The oily residue was crystallized and delivered by washings with anhydrous EtOH and the product was dried over MgSO_4_ (6.1 gr). Yield: 80%. ^1^H-NMR (CDCl_3_): 7.37–7.23 (5H, m, Ar), 5.29–5.18 (1H, m, H18), 4.60–4.48 (2H, 2d, H20), 4.38–4.14 (2H, 2dd, H17,17′), 3.65–3.53 (2H, d, H19), 2.38–2.22 (4H, 2t, H15,15′), 1.68–1.55 (4H, m, H14,14′), 1.38–1.18 (53H, s, H2–13, H2′–13′), 0.94–0.84 (6H, t, H1,1′).


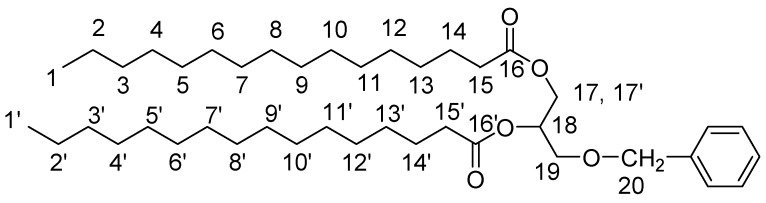


1,2-Palmitoyl-sn-Glycerol

We placed 2.3 gr (3.49 mmol) 3-benzyl-1,2-dipalmitoyl-sn-glycerol in a flask and these were dissolved in 20 mL EtOAc (and some MeOH). Then 230 mg Palladium on Carbon catalyst (Pd-C) (10% *w*/*w*) were added and H_2_ was passed and the reaction mixture was stirred overnight. The Pd-C was the filtered and the mixture was condensed. The product was delivered in acetone and was finally dried (1.79 gr). Yield: 90%. ^1^H-NMR (CDCl_3_): 5.14–5.05 (1H, m, H18), 4.37–4.20 (2H, 2dd, H17,17′), 3.80–3.66 (2H, d, H19), 2.40–2.28 (4H, 2t, H15,15′), 1.68–1.55 (4H, m, H14,14′), 1.38–1.18 (55H, s, H2-13, H2′–13′), 0.94–0.82 (6H, t, H1,1′).


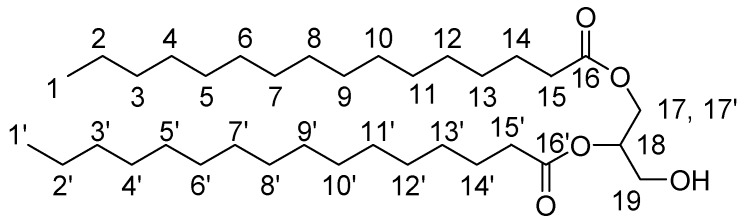


1,2-Dipalmitoyl-sn-Glycerol-3-*O*-Succinic Acid Monoester

We placed 1.025 gr (10.245 mmol) succunic anhydride and 0.250 gr (2.05 mmol) DMAP into a flask and these were dissolved with 16 mL DCM. In this mixture 1.166 gr (2.049 mmol) 1,2-palmitoyl-sn-glycerol was added and the reaction was stirred overnight. The reaction mixture was then extracted with 10% citric acid and the organic layer was washed with H_2_O (×3). The organic layer was delivered and dried with MgSO_4_ and condensed until an oily residue. The final product was delivered after crystallization in anhydrous EtOH (1.36 gr). Yield: 96%. ^1^H-NMR (CDCl_3_): 5.3–5.24 (1H, m, H18), 4.37–4.26 & 4.22–4.12 (4H, 4dd, H17,17′,19,19′), 2.72–2.64 (4H, m, H21,22), 2.35–2.28 (4H,2t, H15,15′), 1.65–1.58 (4H, m, H14,14′), 1.35–1.22 (53H, s, H2–13, H2′–13′), 0.94–0.82 (6H, t, H1,1′).


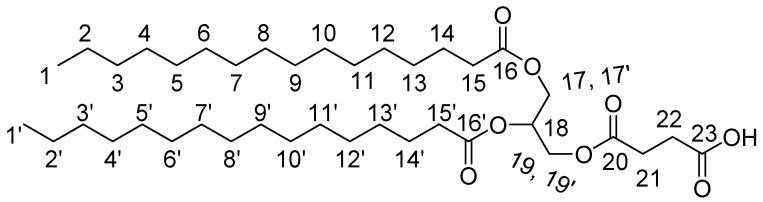


#### 3.2.3. Lipid-BTH

We placed 532.07 mgr (0.78 mmol) of palmitic acid in a flask and this was dissolved in 7.5 mL DCM and the solution was stirred at 4 °C. Then 132.8 μL (0.857 mmol) DIC were added and the mixture was stirred for 15 min. Bis(2-aminophenyl) disulfide dissolved in 500 μL DCM was added and the reaction mixture was stirred overnight. The reaction mixture was sequentially extracted with 15% citric acid (×1) and H_2_O (×3). The organic phase was further dried with Na_2_SO_4_ and condensed until an oily residue was formed. This was dissolved in 6 mL THF/MeOH 5:1 and 34 mg (0.90 mmol) NaBH_4_ were added and the reaction mixture was stirred under Ν_2_ atmosphere for 3 h at rt. Then 515 μL AcOH (9.0 mmol) were added and the mixture was stirred for 1 h under N_2_. Finally the mixture was condensed until an oily residue was formed and the solid product was delivered and recrystallized from anhydrous EtOH (or/and AcCN). The product was finally dried. Total yield: 55%; ESI-MS (M+H)^+^ found: 758.56, calculated: 758.54; ^1^H-NMR (CDCl_3_): 8.02–7.99 (1H, d, H-Ar), 7.86–7.83 (1H, d, H-Ar), 7.50–7.46 (1H, t, H-Ar), 7.41–7.36 (1H, t, H-Ar), 5.28–5.23 (1H, m, H18), 4.38–4.26 & 4.23–4.10 (4H, 4dd, H17,17′,19,19’), 3.5–3.43 (2H, t, H22), 3.04–2.98 (2H, t, H21), 2.32–2.25 (4H, 2t, H15,15′), 1.62–1.55 (4H, m, H14,14′), 1.35–1.20 (58H, s, H2–13, H2′–13′), 0.90–0.86 (6H, t, H1,1′); ^13^C-NMR (CDCl_3_): 173.5, 173 (C16,20), 171.5 (C23), 126, 125, 122.5, 121.5, 115 (C-Ar), 69 (C18), 63, 62 (C17,19), 34 (C15,15′), 32.5 (C21), 32 (C22), 29–30 (C3-13, C3′-13′), 25 (C14,14′), 22.5 (C2,2′), 14 (C1,1′).


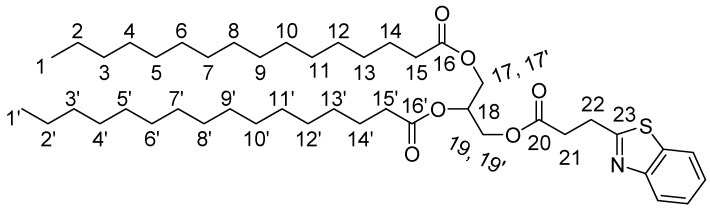


### 3.3. Nanoliposome Preparation

BTH Decorated Nanoliposomes

For the preparation of SUV nanoliposomes incorporating lipid-BTH conjugate, the appropriate amounts of lipids (DPPC, DPPG, DSPE-PEG_2000_-OMe, Chol, and lipid-BTH) were dissolved in a chloroform/methanol (2:1 *v*/*v*) mixture, placed in a round bottom flask and evaporated under vacuum until the formation of a thin lipid film. The lipid film was treated with gas N_2_ and was subsequently connected to a vacuum pump overnight, in order to remove any traces of organic solvent. The lipid film was hydrated with PBS buffer (pH 7.4) at 60 °C [or a 100 mM solution of calcein, prepared in the same buffer, in case of integrity experiments]. After complete lipid hydration and formation of multilamellar liposomes (LMV), the vesicle dispersion was placed under the microtip of a probe sonicator (Sonics & Materials, Inc., Newtown, CT, USA) for 10min, or until the liposome dispersion became completely clear. The nanoliposome dispersion was left in peace for annealing of potential structural defects, at a temperature above the lipid transition temperature for 1–2 h. In case of calcein-encapsulating nanoliposomes, non-encapsulated calcein was removed by size exclusion chromatography.

### 3.4. Characterization of Nanoliposomes

#### 3.4.1. Size Distribution, ζ-Potential, and Stability Studies

Particle size of vesicle dispersions (0.2 mg/mL lipid, in 10 mM PBS pH 7.40) was measured by dynamic light scattering (DLS) technique (Malvern Nano-ZS; Malvern Instruments, Worcestershire, UK) at 25 °C at a 173-degree angle. Zeta potential was measured for the same samples (dispersed in 10 mM PBS, pH 7.40) at 25 °C, by the same instrument (utilizing the Doppler electrophoresis technique). For some of the nanoliposome types, the physical stability (size, polydispersity index and zeta-potential) of the vesicle dispersions (in buffer) was monitored during storage at 4 °C, for a period of 15 days.

#### 3.4.2. Nanoliposome Integrity Studies

The integrity of decorated vesicles was evaluated during incubation of calcein-encapsulating nanoliposomes at 37 °C, in presence and absence of serum proteins (80% *v*/*v*, FCS). Calcein was encapsulated in the vesicles at a concentration (100 mM) at which its fluorescence is quenched. Nanoliposome dispersions (1 mg/mL) were incubated with buffer or FCS, and at various time points, 20 μL samples were drawn for calculation of calcein latency (%). For this, the samples (20 μL) were diluted with 4 mL buffer, pH 7.40 and the fluorescence intensity (FI) was measured (EX 470 nm, EM 520 nm) before and after addition of Triton X-100 at a final concentration of 1% *v*/*v* (that ensures nanoliposome disruption and release of all encapsulated (and latent) dye). Latency (%) was calculated from the equation:

(1) $$\mathrm{Latency}\;\left(\%\right)=100\times \left(\frac{\left[1.1\times Fat\right]-\left[1.1\times Fbt\right]}{\left[1.1\times Fat\right]}\right)$$

where *Fbt* and *Fat* are calcein fluorescence intensities before and after the addition of Triton X-100, respectively.

Integrity (%) was estimated by setting the latency (%) at time zero as integrity 100 (%). At each time point, the integrity (%) during nanoliposome incubation in PBS and FCS was calculated from the equation:

(2) $$\mathrm{Integrity}\;\left(\%\right)=100\times \frac{\mathrm{Latency}\;\left(\%\right)\mathrm{measured}}{\mathrm{Latency}\;\left(\%\right)\mathrm{at}\;\mathrm{time}\;0}$$

#### 3.4.3. Nanoliposome Differential Scanning Calorimetry (DSC) Study

DSC measurements were carried out with a high-sensitivity differential scanning calorimeter DSC Q100 TA Instruments (TA Instruments, New Castle, DE, USA). An aliquot sample of 4 mg of dehydrated nanoliposomes was put into the DSC specimen container. The specimen was scanned at a heating rate of 10 °C/min and the temperature range was from 10 to 80 °C.

## 4. Conclusions

In order to take advantage of the affinity of BTH-groups towards Aβ species [27,28,29,30,31,32,33,34,35,36] and the potential of surface functionalized nanoliposomes for enhanced targeting due to multivalency [14,15,16], we prepared nanoliposomes decorated with BTH. For this, we initially synthesized a lipid-BTH derivative, with high yield, after identifying an optimal methodology. The newly synthesized derivative (lipid-BTH) was incorporated at different amounts (10 and 20 mol%) into nanoliposomes with different lipid compositions, which were characterized for their physicochemical properties, integrity and stability. From all the nanoliposome types evaluated, the pegylated-nanoliposomes (DPPC/Chol/DSPE-PEG_2000_-OMe with 10% lipid-BTH or 20% lipid-BTH nanoliposomes) were the most stable in terms of size stability and membrane integrity. Thus, we conclude that pegylated BTH-nanoliposomes decorated with 10 mol% or 20 mol% BTH, are potential candidate nanoliposome types that deserve further exploitation for the development of improved diagnostic/therapeutic systems for AD.

## Figures and Tables

**Table 1:** Nanoliposome types, molar ratios of composition, and physicochemical characteristics. The vesicle mean diameter (nm), Polydispersity index and ζ-potential (mV) values reported, are the mean values from at least 5 measurements of 3 different preparations, in each case. The % percentage (10% or 20%) of Lipid-BTH is expressed corresponding to DPPC content.

Nanoliposome Type	Molar Ratio	Mean Hydrodynamic Diameter (nm)	Polydispersity Index (PDI)	ζ-Potential (mV)
DPPC/Chol/Lipid-BTH (10%)—**LIP1**	1:1:0.1	84.98 ± 0.22	0.148 ± 0.009	−4.85 ± 0.223
DPPC/Chol/Lipid-BTH (20%)—**LIP2**	1:1:0.2	107.01 ± 0.38	0.318 ± 0.003	−4.13 ± 0.138
DPPC/DPPG/Chol/Lipid-BTH (10%)—**LIP3**	9:1:10:0.1	81.84 ± 0.28	0.186 ± 0.019	−9.61 ± 0.156
DPPC/DPPG/Chol/Lipid-BTH (20%)—**LIP4**	9:1:10:0.2	94.91 ± 1.30	0.186 ± 0.015	−12.2 ± 0.141
DPPC/Chol/DSPE-PEG_2000_-OMe(8%)/Lipid-BTH (10%)—**LIP5**	1:1:0.08:0.1	102.4 ± 0.25	0.170 ± 0.005	−3.13 ± 0.235
DPPC/Chol/DSPE-PEG_2000_-OMe(8%)/Lipid-BTH (20%)—**LIP6**	1:1:0.08:0.2	108.7 ± 1.22	0.168 ± 0.003	−2.27 ± 0.165
